# Development of a Bionic Tube with High Bending-Stiffness Properties Based on Human Tibiofibular Shapes

**DOI:** 10.3390/biomimetics8010018

**Published:** 2023-01-03

**Authors:** Jianqiao Jin, Kunyang Wang, Lei Ren, Zhihui Qian, Xuewei Lu, Wei Liang, Xiaohan Xu, Shun Zhao, Di Zhao, Xu Wang, Luquan Ren

**Affiliations:** 1 Key Laboratory of Bionic Engineering, Ministry of Education, Jilin University, Changchun 130025, China; 2 Weihai Institute for Bionics, Jilin University, Weihai 264402, China; 3 School of Mechanical, Aerospace and Civil Engineering, University of Manchester, Manchester M13 9PL, UK

**Keywords:** bionic design, mechanical bearing-capacity, human tibiofibular, finite-element simulation, healthcare engineering

## Abstract

The human tibiofibular complex has undergone a long evolutionary process, giving its structure a high bearing-capacity. The distinct tibiofibular shape can be used in engineering to acquire excellent mechanical properties. In this paper, four types of bionic tubes were designed by extracting the dimensions of different cross-sections of human tibia–fibula. They had the same outer profiles, but different inner shapes. The concept of specific stiffness was introduced to evaluate the mechanical properties of the four tubes. Finite-element simulations and physical bending-tests using a universal testing machine were conducted, to compare their mechanical properties. The simulations showed that the type 2 bionic tube, i.e., the one closest to the human counterpart, obtained the largest specific-stiffness (*ε* = 6.46 × 10^4^), followed by the type 4 (*ε* = 6.40 × 10^4^) and the type 1 (*ε* = 6.39 × 10^4^). The type 3 had the largest mass but the least stiffness (*ε* = 6.07 × 10^4^). The specific stiffness of the type 2 bionic tube increased by approximately 25.8%, compared with that of the type 3. The physical tests depicted similar findings. This demonstrates that the bionic tube inspired by the human tibiofibular shape has excellent effectiveness and bending properties, and could be used in the fields of healthcare engineering, such as robotics and prosthetics.

## 1. Introduction

After long-term survival-of-the-fittest selection, biological systems produce an optimal balance between structure, shape and function, which gives them a higher degree of natural adaptability. In daily engineering practice, people can extract bionic inspiration from the excellence performance of biological systems, and apply the specifics to the design of products. This kind of bionic optimization of structures has great practical significance in engineering, etc. [1].

The purpose of structural bionics is to study the mechanism of the biological system itself, and reveal the distinct functional and morphological characteristics in order to guide the product design and improve structural performance. The premise of structural bionics is to conduct a detailed study of biological samples to find common characteristics that can be drawn from them in order to improve relevant engineering products [2,3,4]. For instance, Zou et al. [5,6,7,8] used biomimetic methods to design thin-walled tube fittings, which improved axial and transverse energy-absorption by introducing a bamboo-like structure. Xiao et al. [9] investigated the crashworthiness of the horsetail-bionic thin-walled structure (HBTS) under axial dynamic loads, and found that the HBTS with 16 cells had the best crashworthiness. Yin et al. [10] investigated the energy absorption of honeycomb filled tubes, and numerical experiments showed that SEA was a suitable meta-model for honeycomb-filled tubes.

Load-bearing mechanical components such as tubes often have simple shapes, such as prismatic sections. However, modern production methods allow for detailed shape optimization. Therefore, it is now possible to copy some of the complex optimal shapes found in nature and to apply them to everyday engineering components. Zhang et al. [1] designed a bionic tube-fitting based on the leg bone of an ostrich, and compared it with three other tube-fitting designs. Alper et al. [11,12,13,14,15] investigated the load-carrying and energy-absorbing properties of thin-walled structures inspired by Balanus. The results showed that the bionic tube had a lower mass and higher specific-stiffness and structure-efficiency.

In humans, the tibia and fibula not only carry all the body weight but also undergo the acceleration and energy impact generated during movement. Human bones gradually optimized themselves in the long evolutionary process, and the morphological characteristics of tibia–fibula have potential inspirations for load-bearing tubes in engineering. Although the internal mechanism of self-replacement is still not fully clarified, bone tissue thus reduces the risk of fracture and metabolic cost [16,17,18]. Researchers have found that the human tibia has evolved to increase its stiffness and strength and gradually reduce its weight, reducing the body’s energy-expenditure by adjusting its geometry and other aspects [19,20,21,22]. One of the reasons for such a good optimization mechanism is that in the process of bone optimization, the load is more evenly distributed [16,23]. However, local optimization cannot bring significant structural improvement to the whole skeleton, so many scholars have been working for a long time on how to understand the relationship between bone function and morphology [17].

**Figure 1:**
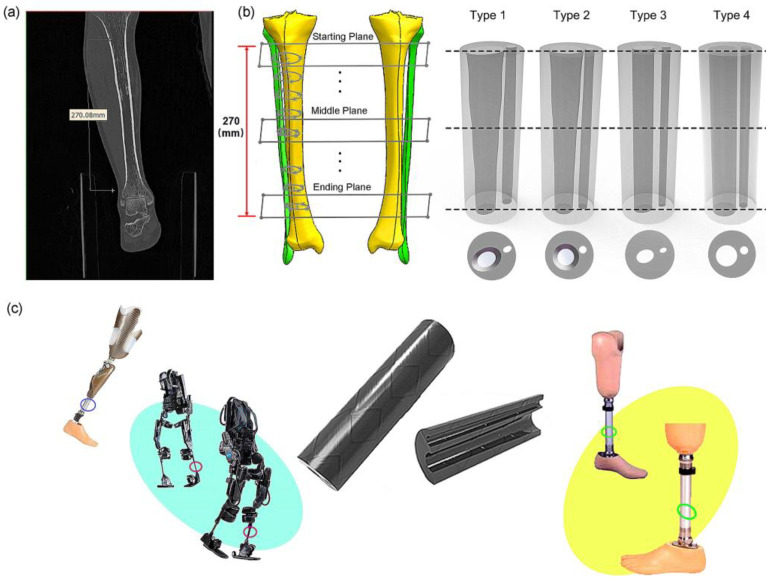
The bionic tube inspired by human tibiofibular bone and its potential applications: (**a**) CT images of human tibia and fibula bones; (**b**) reconstruction of human bones and designs of four bionic tubes; (**c**) potential applications of bionic tubes.

The human tibia primarily plays a load-bearing role in the way it transfers loads between the knee joint and the ankle joint. There are complex load paths in the tibia, because loads must be transferred between the large tibia-plateau into the main shank of the bone and then from the shank of the bone to the large bearing-surface at the ankle joint. In this paper, our interest is in the shape of the shank of the tibia bone. The tibia shank must withstand buckling loads as well as bending loads. This paper focuses on the bending stiffness of the tibia. According to CT scanning images (Figure 1a), the geometric properties of the cross section of the dense bone of the tibia change linearly, that is, it gradually decreases from the proximal end to the distal end [24,25,26,27]. Studies found that the second moment of area and inertia change linearly along the tibia. In addition, osteopathic experiments show that the strain changes at the tibial edge are minimal, which means that the optimized tibial structure has a good resistance to distortion deformation and a strong resistance to bending moment on the sagittal plane [19,28,29,30]. Because the tibia and fibula are subjected to the combined action of torsion, four-point bending and axial extrusion in the process of human movement [31,32,33], the morphological structure of the tibia can effectively bear the load and support the weight of the whole human body. One study found that instead of degeneration, the tibial and fibular structure changes as biological systems renew themselves.

The fibula is not only a load-bearing structure for the lower leg, but it also functions as a linkage mechanism to stabilize the ankle joint [34,35,36]. In conclusion, both the tibia and fibula provide important support for the human body and have good load-bearing performance. This can give useful information in the design of structural-tube applications. Although previous studies have explored other animals, this study focuses on the human tibia and fibula.

Therefore, this paper develops bionic tubes based on the morphological structure of the human tibia shank, which can be applied to the fields of robotics and prosthetics and possibly other fields. The outlines of the human shank were simplified to a regular cylinder, and the inner hollows of the tubes were designed from the irregular shapes of the human tibia–fibula. We first extracted the relevant dominant parameters from the CT images of human tibia and fibula bones, and then designed four types of bionic tubes with the same outer profiles but different inner shapes (Figure 1b). Finally, the specific stiffness was used to evaluate their mechanical properties through both finite-element (FE) simulations and physical bending-tests. In future, several potential applications, such as robotic legs, prosthetics, and human exoskeleton, could be improved by these bioinspired characterizations, in terms of the excellent load-bearing capacity and superior bending-resistance performance of the human tibia–fibula (Figure 1c).

## 2. Materials and Methods

### 2.1. Human Tibiofibular Reconstruction

A healthy male (height: 176.3 cm, age: 30 years old, weight: 75.1 kg) with no history of musculoskeletal disease was selected as the subject to provide tibia–fibula features. The tibia and fibula images were characterized by a 16-row large-aperture spiral CT scanner (Philips, Amsterdam, The Netherlands). The thickness of the scanning layer was s 0.625 mm and the cross-sectional resolutions were 512 × 512 pixels (Figure 1a). The surface model was then reconstructed and smoothed in Mimics (Materialize NV, Leuven, Belgium), and converted to solid models in SolidWorks (Dassault Systèmes SolidWorks Corporation, Waltham, MA, USA). This study was approved by the Ethics Committee of the Second Hospital of Jilin University (Log# 2021072), in accordance with the Declaration of Helsinki (2013) and the Biomedical Research Involving Human Subjects International Code of Ethics for Research (2002).

### 2.2. Design of Bionic Tubes

To design the bionic tubes, we simplified the outlines of the human shank (including the muscles and other soft tissues) to a regular cylinder, and created the inner hollows with irregular shapes based on the outer contour of the human tibiofibular shapes. Eight cross sections of the human tibiofibular bones were obtained to determine four types of bionic tubes with different cross-sectional parameters (Figure 1). The type 1 bionic tube (variable elliptical cross-sections) was directly referenced to the eight cross-sectional profiles of the human tibia–fibula, and each section was fitted to an ellipse. The type 2 tube (variable circular cross-sections) was also based on the eight measurements of the human sample, but replaced the ellipses with equivalent circular cross-sections. The cross sections of type 3 and type 4 tubes were mainly uniform ellipse and circular, respectively. Due to the cost of production in further engineering applications and to ensure the controllability of mechanical property comparison, the human tibiofibular longitudinal curvature was not considered in this study. The centers of different cross-sections of each bionic tube were aligned to a straight line. After the sectional parameters of the four bionic tubes were determined, the methods of lofting and extruding were adopted, to connect the profiles of the different cross-sections, and finally the solid model were created (Figure 1b).

### 2.3. Finite-Element Simulation

To compare the mechanical properties of the four different bionic tubes, finite-element simulations were conducted in ABAQUS (Dassault Systèmes SImulia, Providence, RI, USA). The tubes were meshed by 10-node quadric tetrahedron elements, and the approximate global size control was set as 3 after a sensitivity analysis. Binding constraints fixed by six degrees of freedom were applied at the proximal end of each tube (the variable cross-section fitting was on the larger hole-area side), and coupling constraints were applied at the distal end of the opposite side (coupling the distal cross-sections to the reference point). A concentrated force of 50 N was uniformly applied on the reference point at the distal end [1,37], pointing in the positive direction of the datum axis Z and perpendicular to the length direction of the tubes. In this study, we tested 12 different loading orientations (0°, 30°, 60°, 90°, 120°, 150°, 180°, 210°, 240°, 270°, 300°, 330°) and took the one with weakest bending property to depict.

In the static simulation, the specific-stiffness structural efficiency, *ε*, is defined as the ratio of the elastic modulus to the density of a material multiplied by the deflection [37]. This is used to evaluate the mechanical properties of the four bionic tubes. Specific stiffness is also called the specific elastic modulus, one of the important reference indicators for structural-design materials in engineering. A higher specific-stiffness indicates greater stiffness with the same mass or a lighter material weight with the same stiffness. The specific-stiffness structure efficiency *ε,* in this study, can be calculated as

(1) $$\varepsilon =\frac{E}{\Delta l\cdot m}$$

where *ε* is the specific-stiffness structural efficiency, *E* is the elastic modulus of the material, Δ*l* is the deformation of the distal end, and *m* is the overall mass of the bionic tube. It should be noted that the units of each parameter should be the same as the supporting units in ABAQUS to obtain specific finite-element-analysis data.

### 2.4. Bending Test of Bionic Tubes

The bending tests were conducted by using an electronic universal testing machine (Instron, Canton, MA, USA). To reproduce the operating conditions of the finite-element simulations and facilitate the fixed test on the test machine, the telescopic fixture made of aluminum material was designed and manufactured according to the outlines of the bionic tube. The outer and inner diameters of the cylindrical positioning hole of the fixture were 45 mm and 32 mm, respectively, and the length of the positioning hole was 40 mm. A 50-N compression force was also applied at the distal end of the tubes, and the tests were performed at a constant speed of 3 mm/min. Finally, the bending test of each tube was repeated 10 valid times under same conditions, for further analysis and comparison.

### 2.5. Statistical Analysis

The results of physical experiments are showed as mean ± standard deviation. Statistical significance was tested using ANOVA (single factor) by SPSS 25.0 software (IBM, Armonk, NY, USA). Probability values of *p* < 0.05 were considered statistically significant, and all data are presented at a *p* < 0.05 significance level, unless otherwise stated.

## 3. Results

### 3.1. Division of Bone Parameters and Design of the Bionic Tubes

**Figure 2:**
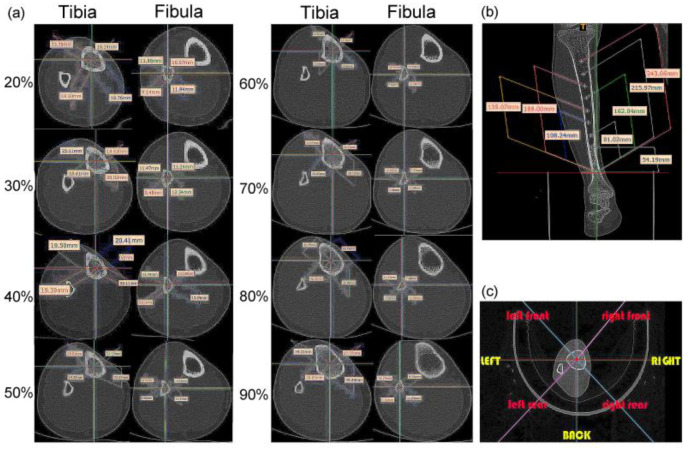
Location and shape selection of human tibiofibular sections: (**a**) size data of the tibia and fibula at different positions; (**b**) overall size division of the calf bone; (**c**) division of eight azimuth points.

During image processing, the tibiofibular bone segment with a total length of 270 mm was determined (the bone-segment sample was selected from the middle of the CT image), and eight positions, at 20%, 30%, 40%, 50%, 60%, 70%, 80% and 90% of the total length were selected [1]. Therefore, the images at sections 54.19 mm, 81.02 mm, 108.24 mm, 135.07 mm, 162.04 mm, 189.00 mm, 215.97 mm, and 243.06 mm were analyzed (Figure 2a,b). Each of these images was extracted and divided according to eight azimuth points: front, back, left, right, left front, left rear, right front, and right rear (Figure 2c).

**Figure 3:**
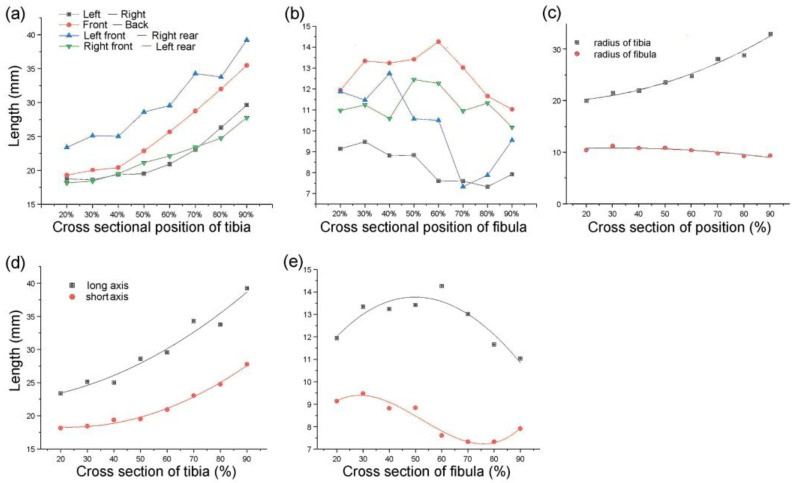
Parameters of tibiofibular section: (**a**,**b**) dimension data of four straight lines at different cross-sections of the tibia (**a**) and fibula (**b**); (**c**) radius-size data of the tibia and fibula at different sections; (**d**,**e**) size data of the longest and shortest lines at different sections of the tibia (**d**) and fibula (**e**).

The distances of the lines from front to back, left to right, left front and right rear, and right front to left back were measured. Each cross-section had four straight lines of different lengths. The tibia and fibula were separately sorted using statistics analysis of these line distances (Figure 3a,b) [1]. The type 1 bionic tube with variable elliptical cross-sections was directly referenced to the sample data of different sections obtained from the above list. The longest of the four lines at each position of the tibia was set as the long axis, *A*, and the shortest line was the short axis, *B*, of the fitting ellipse. Similarly, the long and short axes of the fibula are described as *a* and *b*, respectively (Figure 3d,e).

Accordingly, these four parameters were fitted by polynomials in Equations (2)–(5) as

*A* = 22.13 + 3.13*X* + 16.93*X*^2^ (2)

with determination coefficient *R*^2^ = 0.96,

*B* = 19.42 − 9.68*X* + 20.85*X*^2^, (3)

with determination coefficient *R*^2^ = 0.99,

*a* = 8.72 + 20.98*X* − 23.06*X*^2^ + 2.65*X*^3^ (4)

with determination coefficient *R*^2^ = 0.87,

*b* = 6.01 + 27.01*X* − 64.75*X*^2^ + 41.24*X*^3^ (5)

with determination coefficient *R*^2^ = 0.95, where *X* is the position size of the corresponding bone section.

The type 2 bionic tube was designed with variable circular cross-sections, and the profiles of the tibia and fibula at different positions were extracted. The radii of the tibia and fibula were described as *R* and *r*, respectively. In the solution of the tibial radius *R*, the area formulas of the circle *S*_1_ and ellipse *S*_2_ were set as *S*_1_ *= π · A · B* and *S*_2_ *= π · R*^2^, respectively. The value of *R* equals the square rooting of (*A · B*) when *S_1_* equals *S_2_*. Similarly, the radius of the fibula, *r*, can be calculated as the square rooting of (*a · b*).

Accordingly, the value of *R* and *r* were fitted by polynomials in Equations (6) and (7) as

*R* = 17.67 + 14.33*X* − 14.44*X*^2^ + 19.09*X*^3^ (6)

with determination coefficient *R*^2^ = 0.97,

*r* = 7.08 + 26.20*X* − 51.48*X*^2^ + 27.88*X*^3^ (7)

with determination coefficient *R*^2^ = 0.96, where *X* is the position size of the corresponding bone section.

The design of the type 3 bionic tube was mainly equally elliptical cross-sections, where the long and short axes of the tibia were *A*_1_ = (*A_max_* + *A_min_*)/2 and *B*_1_ *= (B_max_ + B_min_)/*2, respectively. The long and short axes of the fibula were *a*_1_ = (*a_max_* + *a_min_*)/2 and *b*_1_ *= (b_max_ + b_min_)/*2, respectively. These four parameters were calculated as the averages of the maximum and minimum values of the corresponding long and short axes of the tibia–fibula in the type 1 bionic tube. Similarly, the cross sections of the type 4 tube were equally circular, and the radii of the tibia and fibula were set as *R*_1_ and *r*_1_, respectively. Taking the averages of the maximum and minimum radius sizes of tibia–fibula in the type 2 tube, *R*_1_ *= (R_max_ + R_min_)/*2 and *r*_1_ *= (r_max_ + r_min_)/*2 [1] (Figure 3c).

Finally, four types of bionic tubes were designed from the human tibia–fibula bones with different cross-sectional parameters. To facilitate the subsequent manufacture and experimental measurement, the four bionic tubes were uniformly scaled to 1/3, with the same material properties. The final total length and the outer diameter were 90 mm and 30 mm, respectively. The dimensions of each tube are listed in Table 1.

### 3.2. Specifi cStiffness of Bionic Tubes

**Figure 4:**
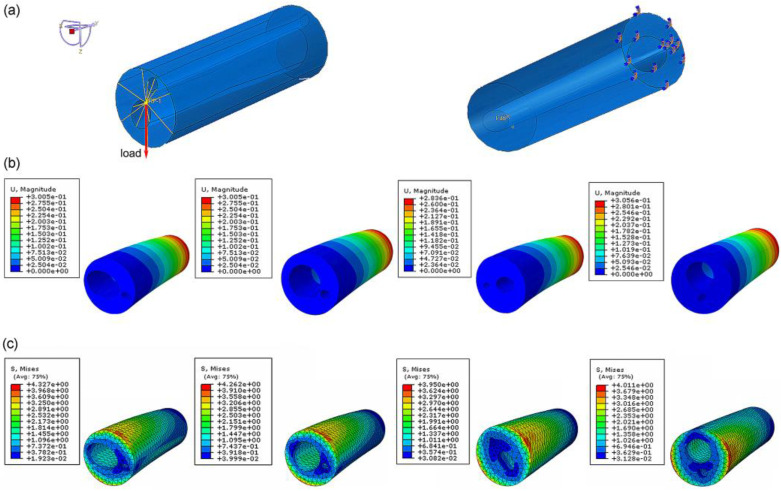
Finite-element analysis of bionic tubes: (**a**) loading conditions and boundary conditions; (**b**) distributions of the spatial displacement; (**c**) distributions of the maximum principal stress.

In the finite-element simulation, the displacement and stress of the four bionic tubes under the same load condition were obtained (Figure 4). The key parameters to quantify the specific stiffness from Equation (1) were listed in Table 2. The results showed that the maximum efficiency *ε*, 6.46 × 10^4^, of the four tubes occurred in type 2, with variable circular cross-sections. The type 1 tube with variable elliptical cross-sections and the type 4 tube with constant circular cross-sections were 6.39 × 10^4^ and 6.40 × 10^4^, respectively, with a very small difference of approximately 0.1%. The lowest value of the specific stiffness was 6.07 × 10^4^ in the type 3 tube with equal-section ellipse. The specific stiffness of the type 2 was approximately 1.1% and 6.43% higher than that of the type 1 and type 3, respectively. This indicated that the type 2 bionic tube had the best specific-stiffness performance, that is, higher stiffness under the same mass or lighter weight under the same stiffness.

### 3.3. Bending Tests of Bionic Tubes

**Figure 5:**
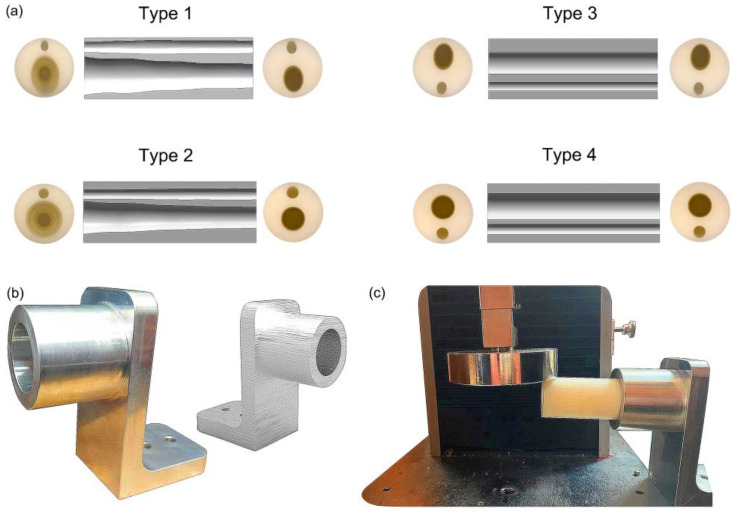
Fabrication and bending test of the bionic tubes: (**a**) cross-sections and inner profiles of the four tubes; (**b**) the telescopic fixture; (**c**) experimental setups.

**Figure 6:**
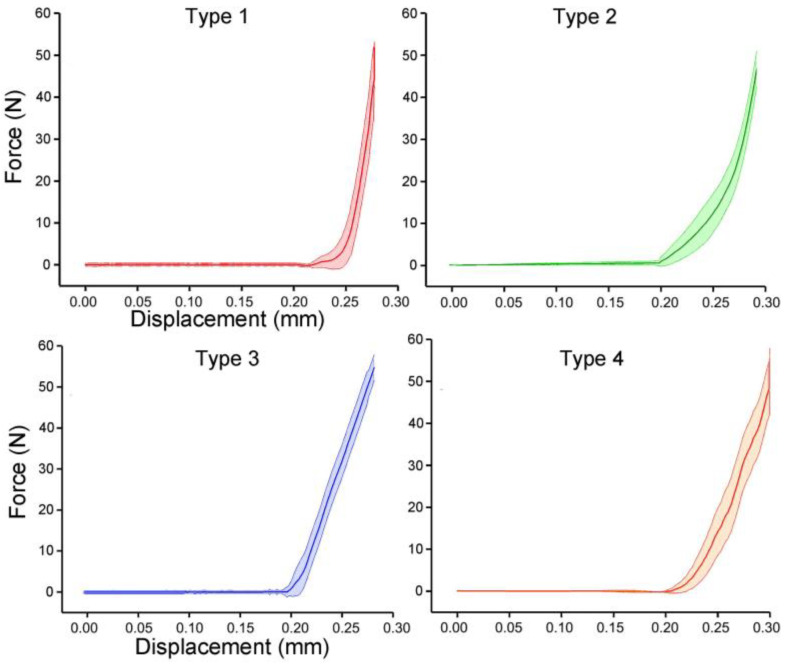
Force-displacement curves of the four bionic tubes. Mean ± standard deviations were depicted, *n* = 10.

**Figure 7:**
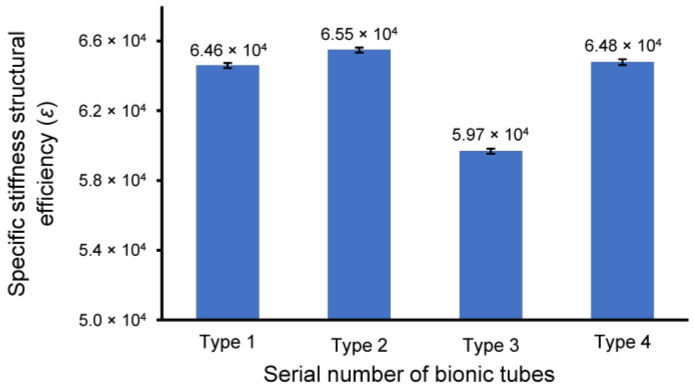
The specific-stiffness structural efficiency, *ε*, of the four bionic tubes. Mean ± standard deviations were depicted, *n* = 10.

The prototypes of the four bionic tubes (Figure 5a) were manufactured from resin materials using a 3D printer J850 (Stratasys, Eden Prairie, MN, USA). The material properties included an elastic modulus of 2370–2650 MPa, a Poisson’s ratio of 0.41, and a density of approximately 1.03 g/cm^3^. During the bending tests, the vertical-lifting tube was in contact with the bending head on the upper part of the electronic universal testing machine (Figure 5b,c). The experimental conditions were the same as in the finite-element simulations. The force-displacement curves of the four bionic tubes were obtained (Figure 6). As there were some small gaps between the fixture and the proximal end of the tube, these curves were relatively flat before the 20-mm displacement. The specific stiffness of each tube was then calculated (Figure 7), according to Equation (1). The results showed that the specific stiffness, *ε*, of the type 2 bionic tube (6.55 × 10^4^) was the maximum, followed by the type 4 (6.48 × 10^4^) and type 1 (6.46 × 10^4^). The lowest specific-stiffness was 5.97 × 10^4^, for the type 3 tube with uniform elliptical cross-sections. These findings were basically consistent with the results from the finite-element analysis.

## 4. Discussion

We used both simulations and physical tests to compare the mechanical properties of four bionic tubes. There are small differences in the stiffness values between the physical experiments and finite-element simulations, especially for the type 4 and the type 3 tubes. In the physical test, the specific stiffness, *ε*, of the type 4 is 8.2% higher than that of the type 3, and this value decreases to 5.4% in the simulations. This might be caused by the fact that the working conditions of the finite-element simulations are slightly different from the physical testing conditions. For example, when a test instrument is unloaded and reloaded during each physical test, the zero load cannot be accurately determined, which will not happen in the simulation.

Although there are some deviations between the finite-element analysis and the physical tests, due to uncontrollable factors, the main findings are the same. The type 2 bionic tube with variable circular cross-sections has the best mechanical properties, followed by the type 4 and type 1. The mechanical properties of the type 3 with uniform elliptical cross-sections is the poorest. Meanwhile, it can also be concluded that the specific stiffness of the type 1 tube increases by 25.8%, compared with that of the type 3, indicating that there is a significant difference between the structural mechanical properties of the optimal tube and the regular tube. This also verifies the fact that the bionic tubes with different structures have diverse mechanical properties under the premise of the controlling variables. Future work may include introducing the curvature of the tibia–fibula into the design of bionic tubes and testing the effects of different materials on the mechanical properties.

## 5. Conclusions

This paper puts forward the function and significance of the human tibiofibular bone, and proves that the shape of the tibia–fibula can be parameterized in the form of bionics, to guide the design of engineering tubes. Specifically, the distinct features of the human tibia–fibula were analyzed. The cross-sections at different positions along the human tibiofibular length were divided into eight directions. By connecting their position between two points at each cross section, four lines were formed through analysis of their length, allowing the corresponding calculation criteria to provide reasonable profile data on the selected target size. Then, four types of bionic tubes were designed with same outer profiles but different inner shapes, according to the parameters obtained. The structural efficiency of the specific stiffness was introduced as a reference, to compare the mechanical properties of different tubes by using both finite-element simulations and physical bending-tests. The results indicated that there were prominent differences in the mechanical properties of bionic tubes with different structures and designs. With proper determination of the key parameters, the specific stiffness of the bionic tube could be increased by as much as 25.8%. In future, the bionic tubes can be applied to the field of healthcare engineering, for use with walking-assisted devices, prosthetics, and human exoskeletons.

## Figures and Tables

**Table 1:** Key design-parameters of the four bionic tubes.

Type	Fitting Parameter	Cross-Sectional Position							
		20%	30%	40%	50%	60%	70%	80%	90%
1	long axis of the tibia (mm)	23.39	25.11	25.01	28.61	29.56	34.27	33.75	39.22
	short axis of the tibia (mm)	18.14	18.43	19.39	19.52	20.92	23.05	24.75	27.77
	long axis of the fibula (mm)	11.94	11.94	13.24	13.42	14.26	13.02	11.66	11.03
	short axis of the fibula (mm)	9.14	9.14	8.82	8.84	7.61	7.33	7.33	7.92
2	radius of the tibia (mm)	20.02	21.51	22.02	23.63	24.87	28.11	28.90	33.01
	radius of the fibula (mm)	10.45	11.25	10.81	10.89	10.42	9.77	9.24	9.35
3	long axis of the tibia (mm)	31.31							
	short axis of the tibia (mm)	22.96							
	long axis of the fibula (mm)	12.65							
	short axis of the fibula (mm)	8.41							
4	radius of the tibia (mm)	26.51							
	radius of the fibula (mm)	10.25							

Type 1 cross-sections: variable ellipses; type 2 cross sections: variable circles; type 3 cross sections: uniform ellipse; type 4 cross sections: uniform circles. All types of the bionic tubes are 90 mm in total length and 30 mm in outside diameter.

**Table 2:** Finite-element analysis of the bionic tubes.

Bionic Tube	Axial Displacement in the Distal End (mm)	Mass (kg)	Specific Stiffness *ε*
type 1	2.96 × 10^−1^	5.32 × 10^−2^	6.39 × 10^4^
type 2	2.94 × 10^−1^	5.31 × 10^−2^	6.46 × 10^4^
type 3	2.78 × 10^−1^	5.98 × 10^−2^	6.07 × 10^4^
type 4	3.05 × 10^−1^	5.16 × 10^−2^	6.40 × 10^4^

## Data Availability

The datasets generated during and/or analyzed during the current study are available from the corresponding author on reasonable request.
